# Supplementary material for: WordCommentsAnalyzer: A windows software tool for qualitative research
Source: F1000Res. 2018 Sep 4;7:536. Originally published 2018 May 3. [Version 2] doi: 10.12688/f1000research.14819.2 (PMC6137411; doi:10.12688/f1000research.14819.2)
Supplement: Supplementary file 3 [file f1000research-7-17614-s0002.tgz › b2a79496-4ada-47c4-9682-a99ce867836d.zip!/Eight Iranian Health Journals/The Journal of Toloo-e-Behdasht_2.docx]

© The Journal of Toloo-e-Behdasht. This work is licensed under CC BY 4.0 (https://creativecommons.org/licenses/by/4.0/).

Volume & Issue: دوره 16 - شماره 6

Title: بررسی مقایسه ای میزان آلودگی توت فرنگی به استافیلوکوک اورئوس، اشریشیاکلی و سایر کلی فرم ها در استان کردستان

http://tbj.ssu.ac.ir/article-1-2498-fa.html

مقدمه: کردستان از جمله استان های مهم در زمینه تولید توت فرنگی در کشور محسوب می شود. در کشت سنتی به علت تماس دست و استفاده از کودهای حیوانی در مزارع و آلوده شدن خاک، انتقال باکتری های پاتوژنی چون استافیلوکوک اورئوس و اشریشیاکلی و سایر کلیفرم ها به آسانی توسط میوه ها و سبزیجات این مزارع صورت می گیرد.هدف از این تحقیق، بررسی میزان آلودگی به استافیلوکوک اورئوس و اشریشیاکلی و سایر کلیفرم ها در انواع توت فرنگی تولید شده در استان کردستان بوده است. روش بررسی: در یک مطالعه توصیفی، در سال1393، تعداد 150 نمونه توت فرنگی شامل 90 نمونه کشت مزرعه(سنتی)، 30 نمونه گلخانه ای و 30 بسته بندی سطح شهر سنندج مورد بررسی قرار گرفت. طبق دستور العمل استاندارد ایران، نمونه ها از نظر وجود استافیلوکوکوس اورئوس به شماره  1194، شمارش کلی به شماره  5272، شمارش کلی فرم به شماره  9236 و شناسایی اشریشیاکلی به شماره  2964 انجام شد. یافته ها: از 150 نمونه مورد بررسی، 16 نمونه (%66/10) به استافیلوکوکوس اورئوس کواگولاز مثبت آلوده بودند. بیشترین آلودگی در توت فرنگی بسته بندی مشاهده شد. درخصوص حضور کلیفرم ها در توت فرنگی،از 150 نمونه مورد بررسی، 30 نمونه (%20) به کلیفرم ها آلوده بودند.درخصوص حضور اشریشیاکلی، از 150 نمونه مورد بررسی، 21 نمونه (%14) به اشریشیا کلی آلوده بودند. نتیجه گیری: نتایج ما نشان داد، این میوه به دلیل استفاده از کودهای حیوانی و انسانی و استفاده از آب های سطحی جهت آبیاری و شستن توت فرنگی با آب قبل از مصرف می تواند در کاهش آلودگی تاثیر داشته باشد.

Title: کاربرد مدل کلاس پنهان بیز در تعیین ارزش تشخیصی SPECT و MRI مغز جهت تشخیص حس بویایی بعد از تروما بدون حضور استاندارد طلایی

http://tbj.ssu.ac.ir/article-1-1278-fa.html

چکیده مقدمه: حس بویایی کیفیتی غیرقابل توصیف به زندگی انسان می بخشد. اختلال در این حس در زندگی روزمره مشکلات زیادی ایجاد خواهد کرد. MRI و SPECT دو روش تعیین حس بویایی است که هیچ کدام استاندارد طلایی نیستند. مدل کلاس پنهان بیز روشی صحیح جهت تعیین ارزش تشخیصی این آزمون ها می باشد. روش بررسی: دو آزمون MRI و SPECT برروی 63 بیمار حائز شرایط مورد مطالعه که از ابتدای تیرماه 1390 تا ابتدای مهر 1391به بیمارستان شهید رهنمون یزد مراجعه کردند، انجام شد. نتایج حاصل به عنوان تابع درستنمایی ماکزیمم با توزیع پیشین، بااستفاده از زنجیر مارکوف مونت کارلو در نرم افزار winbugs نسخه 3 . 4 . 1 ترکیب شده و میانه ی توزیع پسین به عنوان برآورد پارامترها معرفی شد.دو مدل وابسته و مستقل شرطی با استفاده از معیار انحراف اطلاعات (DIC) مقایسه شد. یافته ها: حساسیت و ویژگی MRI جهت تشخیص بویایی غیرنرمال، در مدل وابسته ی شرطی، 58% و 89%  و برای SPECT به ترتیب 73% و 84% تعیین و ارزش اخباری مثبت و منفی هردو تست نیز برآورد شد. همگرایی زنجیر و نیکویی برازش مدل نیز با استفاده از نمودارهای سری زمانی وجلمن-روبین و p-value بیز تأیید شد. با توجه به کوچکتر بودن DIC برای مدل وابسته، این مدل جهت برازش به این داده ها برتر از مدل مستقل تعیین شد. نتیجه گیری: در مدل کلاس پنهان بیز، نتایجی متفاوت از حالتی که از این مدل استفاده نشده، حاصل می گردد. بهتر است هردو مدل وابسته و مستقل شرطی  به داده ها برازش شده و نهایتاً با هم مقایسه شوند.

Title: بررسی تطبیقی برنامه درسی دوره دکترای تخصصی آموزش بهداشت و ارتقاء سلامت در ایران و دانشگاه های برتر جهان

http://tbj.ssu.ac.ir/article-1-2255-fa.html

مقدمه: در نظام های آموزشی نوین و کارآمد مطالعات تطبیقی یکی از شیوه های همگام شدن با تحولات و پیشرفت های جدید از طریق شناسایی نوآوری ها می باشد. مطالعه حاضر با هدف ارائه پیشنهادات کاربردی جهت بهبود سرفصل های تدریس شده رشته آموزش بهداشت و ارتقاء سلامت در ایران، انجام شد. روش بررسی: این پژوهش با روش تطبیقی به کمک الگوی بردی و نیز روش توصیفی- تحلیلی انجام شده است. گردآوری داده ها با استفاده از اطلاعات موجود در سایت صد دانشگاه برتر جهان در حوزه سلامت عمومی صورت پذیرفت. یافته ها: در بین دانشگاه های  مورد بررسی، رشته آموزش بهداشت و ارتقاء سلامت در هفت دانشگاه تدریس      می شد. دروسی هم چون اصول اپیدمیولوژی، رفتار شناسی، روش های آماری، مبانی آموزش و ارتقاء سلامت، مشکلات سلامتی نوظهور، ارتقاء سلامت از طریق علوم رفتاری، تعیین کننده های سلامت جامعه، باورهای بهداشتی در فرهنگ های مختلف، روش تحقیق کیفی جزو دروسی هستند که بطور تخصصی اجباری در دانشگاه های ذکر شده تدریس می شوند، ولی این دروس در واحدهایی که تحت عنوان دروس تخصصی اجباری و اختیاری در ایران تدریس می شوند، وجود ندارد . هم چنین دروس ارتقاء سلامت اجتماع محور، و کارآموزی در عرصه که جزو دروس اجباری در ایران می باشند، در هیچ کدام از دانشگاه های مورد بررسی تدریس نمی شوند. نتیجه گیری: بنظر می رسد دروس روش های آماری در علوم سلامتی و اصول اپیدمیولوژی که در تمامی     دانشگاه های بررسی شده تدریس می شوند، می بایستی در برنامه درسی دوره ی دکترا گنجانده شوند.

Title: بررسی اختلالات چربی خون و برخی عوامل مرتبط با آن در مبتلایان به اسکیزوفرنی و اختلال دو‌قطبی در شهرستان یزد

http://tbj.ssu.ac.ir/article-1-2417-fa.html

مقدمه: میزان‌های میرایی در مبتلایان به اسکیزوفرنی و اختلال دوقطبی بیشتر از جمعیت عمومی است. از مهم ترین علل طبیعی مرگ در این بیماران بیمار‌ی‌های عروق قلب است. هدف از این مطالعه بررسی شیوع اختلالات چربی خون و برخی عوامل مرتبط در بیماران مذکور می‌باشد. روش بررسی: در این مطالعه که به صورت مقطعی تحلیلی انجام شد،200 بیمار در دوگروه مبتلا به اسکیزوفرنی و اختلال دوقطبی مورد بررسی قرار گرفتند. شیوع اختلالات چربی خون و برخی عوامل خطر مرتبط با آن به تفکیک برای هر دو گروه محاسبه گردید. برای توصیف داده‌ها از آمار توصیفی        (میانگین و انحراف معیار)، برای مقایسه میانگین‌ها ازآزمونt  مستقل و جهت مقایسه‌ی شیوع‌ها از آزمون کای اسکوئر استفاده شد. یافته‌ها: شیوع اختلال چربی خون در بیماران اسکیزوفرن 57 % و در مبتلایان به اختلال دوقطبی72 % بود(027/0p ). در بین اختلالات چربی خون، اختلال HDL-C پایین به ترتیب با شیوع 54 % و 61 % در بیماران اسکیزوفرن و دو قطبی دارای بیشترین شیوع بود(562/0 p ). از میان عوامل خطرزای مرتبط با اختلالات چربی خون، در بیماران اسکیزوفرن مصرف سیگار با شیوع 38 % و در مبتلایان به اختلال دوقطبی چاقی شکمی با شیوع 48 % بیشترین شیوع را داشت. نتیجه‌گیری: نتایج این تحقیق حاکی از شیوع بالای اختلالات چربی خون و عوامل خطر مرتبط با آن در مبتلایان به اسکیزوفرنی و اختلال دوقطبی است و در خصوص این عارضه لزوم توجه بیشتر به این بیماران و فوریت اقدامات مداخله ای را یادآوری می‌کند.

Title: طراحی برنامه درسی بهداشت بلوغ ویژه دختران دوره تحصیلی متوسطه اول در نظام آموزش و پرورش ایران

http://tbj.ssu.ac.ir/article-1-2334-fa.html

مقدمه: بلوغ مرحله مهمی از زندگی است که وجود برنامه درسی ویژه آن، برای آگاهی نوجوانان ضروری است. هدف آموزش بهداشت بلوغ، کمک به نوجوانان برای اتخاذ و حفظ رفتارهای سالم است. در این راستا، هدف پژوهش حاضر، طراحی برنامه درسی بهداشت بلوغ ویژه دختران دوره تحصیلی متوسطه اول در ایران تعیین شد. روش بررسی: این پژوهش توصیفی به روش زمینه یابی انجام شد. جامعه مورد بررسی شامل متخصصان برنامه ریزی درسی، کارشناسان بهداشت علوم پزشکی و همچنین کارشناسان بهداشت مدارس شهر یزد بودند. نمونه گیری به شیوه سرشماری انجام شد که مجموعاً تعداد 28 نفر را شامل شد. ابزار پژوهش، پرسشنامه محقق ساخته بود که روایی صوری و محتوایی آن تعیین شد. پایایی ابزار از طریق آلفای کرونباخ عدد 90/0 به دست آمد. داده ها از طریق نرم افزار SPSS با استفاده از شاخص های مرکزی و تی تک نمونه ای تحلیل شدند. یافته ها: نتایج این مطالعه نشان داد برنامه درسی بهداشت بلوغ در بخش های اهداف، محتوا، روش تدریس و روش ارزشیابی دارای مولفه های تعیین کننده است. همچنین اعتبار طرح تدوین شده، از دیدگاه کارشناسان با سطح 99 درصد اطمینان تایید شد. نتیجه گیری: از آنجا که بسیاری از والدین به دلیل موانع فرهنگی از ارائه توضیحات جامع درباره بلوغ و مسائل آن برای فرزندانشان خودداری می کنند، وجود برنامه درسی در این زمینه اهمیت دو چندان می یابد. در پژوهش حاضر، چارچوب برنامه درسی بهداشت بلوغ طراحی و تایید شد و در قالب درس مستقل ویژه دختران دوره تحصیلی متوسطه اول پیشنهاد گردید.

Title: بررسی میزان فرسودگی شغلی و عوامل مرتبط با آن در کارگران صنعت نساجی:مطالعه موردی در شهر یزد

http://tbj.ssu.ac.ir/article-1-2402-fa.html

مقدمه : یکی از مسایلی که منابع انسانی را امروزه تهدید می کند، فرسودگی شغلی  بوده و در صور تی که فرد دچار ا ین حالت شود، تغییرات منفی در  نگرش ، روحیه و رفتار او ایجاد ،و  از لحاظ هیجانی دچار خستگی شده و به تدر یج در ایفای وظایفش احساس کاهش شا یستگی می کند.با توجه به نقش مهم کارگران در تولید بر آن شدیم مطالعه ای با هدف« بررسی میزان فرسودگی شغلی و عوامل موثر بر آن  در کارگران یکی از صنایع نساجی شهر یزد» انجام دهیم روش بررسی :در این پژوهش توصیفی -تحلیلی جامعه  آن  200 نفر از کارگران شرکت نساجی بودند، که به روش نمونه گیری طبقه ای انتخاب شدند.ابزار جمع آوری داده ها پرسشنامه مشخصات جمعیت شناختی  وپرسشنامه استاندارد 22 سوالی فرسودگی شغلی مازلاک بود که داده ها بعد از کدگذاری با استفاده از نرم افزارSPSS نسخه 16و آمار توصیفی و آنالیز واریانس و T-test و ضریب همبستگی تجزیه و تحلیل گردید . یافته ها: میانگین نمره فرسودگی شغلی کارگران 88/24±2/52( از طیف 132-0) بود که در سطح فرسودگی شغلی متوسط قرار دارد. در این مطالعه 59 درصد از کارگران دارای کفایت فردی پایین، 52 درصد  خستگی هیجانی شدید و 25 درصد دارای مسخ شخصیت در سطح شدید بودند. بین فرسودگی و ابعاد آن همبستگی معنی داری وجود داشت (001/=p) همچنین بین فرسودگی شغلی با تحصیلات ،وضعیت تاهل ،سابقه کار و رضایت از درآمد ، ارتباط معنی داری دیده شد(05/ ≥ p ) ولی بین تعداد فرزند و بیماری ارتباط معنی دار نبود. نتیجه گیری: با توجه به بالا بودن فرسودگی شغلی و ابعاد آن در کارگران  واثرات منفی آن بر عملکرد و سلامت روانی آنها توصیه می گردد کلاس های آموزشی و کارگاه های مهارتی برای آنها برگزار گردد و ضمناً مدیریت به خواسته های آنها توجه بیشتر نمو ده و مشارکت آنها را در برنامه ها جلب نماید.

Title: بررسی نقش نگرش، هنجارهای انتزاعی و کنترل رفتاری درک شده در پیش بینی مصرف شیر در دانشجویان دانشگاه علوم پزشکی یزد

http://tbj.ssu.ac.ir/article-1-2185-fa.html

مقدمه: اثرات مثبت مصرف شیر در جوانان شامل افزایش تراکم استخوانی، کاهش بروز بیماری های قلبی عروقی،  دیابت و  بهبود نمایه توده بدنی است. با این حال کمتر از 40 درصد از جوانان 29-20 ساله روزانه مقادیر کافی از شیر را دریافت می کنند. مطالعه حاضر به منظور بررسی نقش نگرش، هنجارهای انتزاعی و کنترل رفتاری درک شده در پیش بینی مصرف شیر در دانشجویان دانشگاه علوم پزشکی یزد صورت پذیرفت. روش بررسی: این مطالعه مقطعی بر روی 385 دانشجوی دانشگاه علوم پزشکی یزد که به روش چند مرحله ای انتخاب شده بودند انجام گرفت. ابزار گردآوری اطلاعات شامل پرسشنامه محقق ساخته در دو بخش اطلاعات دموگرافیکی و سازه های تئوری رفتار برنامه ریزی شده بودکه روایی و پایایی آن سنجیده شده بود. داده های جمع آوری شده  با استفاده از نرم افزار18 SPSS و آزمون های آمار توصیفی، آزمون همبستگی اسپیرمن و رگرسیون چند متغیره مورد تجزیه و تحلیل قرار گرفتند. یافته ها:  دامنه سنی دانشجویان شرکت کننده در مطالعه 18 تا 38 سال بود. از مجموع 385 دانشجوی شرکت کننده در مطالعه 2/65 درصد دختر و 8/34 درصد پسر بودند.در این مطالعه سازه های نگرش، هنجارهای انتزاعی و کنترل رفتاری درک شده توانستند 14 درصد از واریانس قصد را پیش بینی کنند( 14/0=R2 ,  79/20=, F001/0>p). همچنین سازه های قصد و کنترل رفتاری درک شده توانستند 24 درصد از واریانس رفتار مصرف شیر را توضیح دهند( 24/0=R2 , 76/30=, F001/0>p). نتیجه گیری: سازه های تئوری رفتار برنامه ریزی شده یک چهارچوب نظری مناسب برای مطالعه رفتار مصرف شیردر دانشجویان است و می تواند در مداخلات برنامه ریزی شده برای ارتقاء سلامت دانشجویان به کار گرفته شود.

Title: تاثیر آموزش مهارت های زندگی بر میزان رضایت زناشویی متقاضیان طلاق در مرکز تحکیم بنیان خانواده شهر یزد

http://tbj.ssu.ac.ir/article-1-2397-fa.html

مقدمه: یکی از آسیب های روابط زناشویی کاهش میزان رضایتمندی زناشویی است که می تواند بنیاد زندگی مشترک را در خطر قرار دهد.این پژوهش با هدف تاثیر آموزش بر میزان رضایت زناشویی افراد متقاضی طلاق مراجعه کننده به مرکز تحکیم خانواده شهر یزد انجام شد. روش بررسی: مطالعه حاضر مداخله ای از نوع پیش ازمون و پس ازمون بود.  نمونه های به صورت در دسترس و با جایگزین تصادفی به تعداد 76 نفر از بین مراجعان به مرکز تحکیم خانواده انتخاب شدند. شرکت کنندگان که روال معمول آموزش مرکز را طی دو جلسه 3 ساعته شامل مباحث مختلف آموزش مهارت های زندگی بود، در مرکز گذراندند و پرسشنامه رضایت زناشویی را قبل و بعد از دوره اموزشی تکمیل نمودند. در این پژوهش از پرسشنامه رضایت زناشویی انریچ و سوالات مربوط به ویژگی های دموگرافیک استفاده شد. داده ها با استفاده از نرم افزار16SPSS  وآزمون های کای آسکوار و ویل کاکسون تجزیه وتحلیل گردید. یافته ها: میانگین سنی زوجین 39/6 ± 69/29 بود و میانگین نمره  رضایت زناشویی قبل از آموزش در بین شرکت کنندگان 12/26± 25/1و بعد از آموزش 55/26± 25/1 اعلام شد که تفاوت معنادار آماری را نشان نداد. بین میانگین نمره میزان رضایت زناشویی قبل و بعد از دریافت آموزش بر حسب متغیرهای دموگرافیک مثل سطح تحصیل ، میزان درآمد ، محل سکونت ، سن وجنس نیز تفاوت معناداری مشاهده نشد ولی تغییراتی درنمرات قبل و بعد آموزش وجود داشت. نتیجه گیری: بنظر می رسد شرایط خاص مراجعانی که برای طلاق اقدام نموده اند به گونه ای است که تاثیر آموزش مهارت های زندگی نتوانسته تغییر معناداری را قبل و بعد از دریافت آموزش بر سطح رضایت مندی زناشویی ایجاد  نماید. لازم است با بررسی های بیشتر دلایل کم بودن تاثیر این مداخله مشخص گردد که آیا آموزش بر رضایت زناشویی تاثیر ندارد یا باید برنامه هایی برای افزایش کارایی این دوره ها طراحی گردد.

Title: بررسی میزان مصرف چربی براساس الگوی اعتقاد بهداشتی در زنان شهر یزد در سال 94

http://tbj.ssu.ac.ir/article-1-2132-fa.html

مقدمه:. یکی از ارکان سلامت جامعه، تامین نیازهای جسمی و ذهنی از طریق تغذیه مطلوب است. با توجه به اینکه جمعیت های مختلف ، عادات غذایی و بیماری های مزمن متفاوتی دارند دستورالعمل مصرف چربی در بین آنها متفاوت است. سطوح سرمی کلسترول عامل خطر عمده بیماری های قلبی و عروقی است . هدف از این مطالعه تعیین کاربرد مدل اعتقاد بهداشتی بر مصرف چربی در زنان شهر یزد در سال 1394 انجام شد.   روش بررسی: این مطالعه توصیفی تحلیلی به صورت مقطعی بر روی زنان شهر یزد در سال ۹۴ انجام شد. تعداد نمونه203 نفر از زنان تحت پوشش مراکز بهداشتی بودند که به صورت تصادفی دو مرحله ای انتخاب شدند. ابزار گردآوری داداه ها شامل دو پرسشنامه  بود پرسشنامه اول ، پرسشنامه محقق ساخته بود و پرسشنامه دوم نسخه اصلاح شده از پرسشنامه بسامد خوراک ۱۶۸ قلم برای مطالعه قند و لیپید تهران بود که براساس ضائقه مردم یزد 10 ماده غذایی به آن اضافه شده بود.تجزیه وتحلیل داده ها توسط نرم افزارspss و با استفاده از شاخص های مرکزی وپراکندگی،ضریب همبستگی اسپیرمن،رگرسیون خطی و آنالیز واریانس صورت گرفت. یافته ها: میانگین امتیازات سازه های مدل اعتقاد بهداشتی شامل حساسیت درک شده ۲۲/۴±۹۳/۱۴ ، شدت درک شده ۰۷/۳±۰۲/۱۲ ، منافع درک شده۷۹/۱± ۰۹/۱۷، موانع درک شده۴۴/۵ ±۳۲/۱۸، راهنمای عمل ۴۶/۱±۰۶/۳، خودکارآمدی۹۳/۴± ۱۶/۲۳ بود. میانگین مقدار چربی دریافتی از رژیم غذایی روزانه ۶۲/۴۸± ۲۵/۹۷گرم بود.متوسط مقدار کالری دریافتی (Kcal)۸/۱۱۵۶±۶۱/۲۵۴۸ بود. براساس آنالیز رگرسیون لوجستیک هیچکدام از سازه های الگوی اعتقاد بهداشتی پیش بینی کننده قوی برای مصرف چربی نبودند. نتیجه گیری:. نظر به اینکه سازه های الگوی اعتقاد بهداشتی انجام رفتار مصرف چربی را پیش بینی نمی کردند میتوان نتیجه گیری کردکه الگوی فوق در تبیین رفتار مصرف چربی کارآمد نبوده است و میتوان از الگوهای دیگر برای بررسی رفتار مصرف چربی استفاده کرد .

Title: ارزیابی میزان انتقال فلزات روی، کادمیوم و نیکل در شیر سویای تهیه شده با روش‎های مختلف و انتقال آن به پنیر سویا

http://tbj.ssu.ac.ir/article-1-1848-fa.html

مقدمه: آگاهی و نگرانی در مورد آلودگی خاک با فلزات سنگین و اثرات نامطلوبی آن‎ها در زنجیره غذایی، در حال افزایش است. امروزه فلزات سنگین از مهمترین منابع آلوده کننده می باشند و مشکل جدی برای سلامت عموم محسوب می شوند. مصرف غلات یکی از منابع عمده دریافت این گونه عناصر است، که سویا نسبت به دیگر محصولات قادر به تجمع بیشتری از این مواد است. روش بررسی: در این مطالعه سعی شد میزان سه عنصر فلزی روی، کادمیوم و نیکل در محصولات حاصل از سویا اندازه‎گیری شود. در ابتدا شیر سویا و پنیر سویا با استفاده از روش‎های معمول تهیه گردید و پس از هضم نمونه ها، به منظور سنجش این عناصر از دستگاه ICP-OES استفاده شد. سپس انتقال میزان فلزات در تمامی روش‎های تهیه محصولات سویا مورد مقایسه قرار گرفت. یافته‎ها: در مورد فلز روی، شیر سویاهای تهیه شده با روش سریع دارای مقادیر بالاتری (05/0P<) روی بودند و در میان پنیرها، میزان روی در پنیرهای کلسیمی حاصل از هر سه روش تهیه شیر، به طور معنی‎داری بالاتر  (05/0P<)بود. میزان نیکل در شیر و پنیرهای روش سریع بالاتر  (05/0P<)از بقیه روش‎ها بوده است. در روش سنتی مقادیر زیادی نیکل از طریق خیساندن دانه و اکارا از دسترس خارج شده است. در اندازه‎گیری‎ها مشخص شد که شیرهای تهیه شده با روش سریع و پنیرهای حاصل دارای مقادیر قابل توجهی از کادمیوم  (05/0P<) بودند، اما در بقیه روش‎ها مقدار کادمیوم پایین‎تر از حد قابل تشخیص بود. نتیجه‎گیری: براساس نتایج بدست آمده، نوع روش تهیه محصولات سویا و خیساندن دانه سویا می‎تواند روی میزان انتقال فلزات و خارج‎سازی این فلزات نقش موثری داشته باشد. از طرفی نوع لخته تشکیل شده در پنیرها، در تجمع فلزات هم اثر قابل توجهی داشته است.
